# The P21-Activated Kinase 1 and 2 As Potential Therapeutic Targets for the Management of Cardiovascular Disease

**DOI:** 10.53941/ijddp.v1i1.179

**Published:** 2022-12-21

**Authors:** Honglin Xu, Dingwei Wang, Chiara Ramponi, Xin Wang, Hongyuan Zhang

**Affiliations:** Michael Smith building, Division of Cardiovascular Sciences, Faculty of Biology, Medicine and Health, The University of Manchester, Manchester, UK

**Keywords:** Group I p21-activated kinases, cardiovascular disease, homeostasis

## Abstract

Group I p21-activated kinases (Paks) are members of the serine/threonine protein kinase family. Paks are encoded by three genes (Pak 1 − 3) and are involved in the regulation of various biological processes. Pak1 and Pak2 are key members, sharing 91% sequence identity in their kinase domains. Recent studies have shown that Pak1/2 protect the heart from various types of stresses. Activated Pak1/2 participate in the maintenance of cellular homeostasis and metabolism, thus enhancing the adaptation and resilience of cardiomyocytes to stress. The structure, activation and function of Pak1/2 as well as their protective roles against the occurrence of cardiovascular disease are described in this review. The values of Pak1/2 as therapeutic targets are also discussed.

## 1 Introduction

The P21-activated kinase (Pak) family is a group of kinases that plays crucial roles in multiple biological processes. The first kinase of the Pak family was isolated from the rat brain in 1994 and was found to be one of serine/threonine protein kinases activated by Cdc42 and GTP-bound Rac1 [1]. To date, six Pak isoforms (Pak1–6) have been identified. Pak proteins are classified into group I (Pak1 to Pak3) and group II (Pak4 to Pak6) depending on their sequence homology [2–5]. The expression of some isoforms is tissue-specific, whereas others are ubiquitously expressed. Pak protein isoforms perform numerous functions. Importantly, Pak1 and Pak2 regulate numerous cardiac functions [6,7]. In this review, the basic structure and the mechanism underlying the activation of Pak1 and Pak2 are discussed. How these two proteins regulate various signalling pathways, their involvement in cardiovascular disease, and their potential to be therapeutic targets are also reviewed.

### 1.1 Pak1/2 Structure and Activation

**Figure 1:**
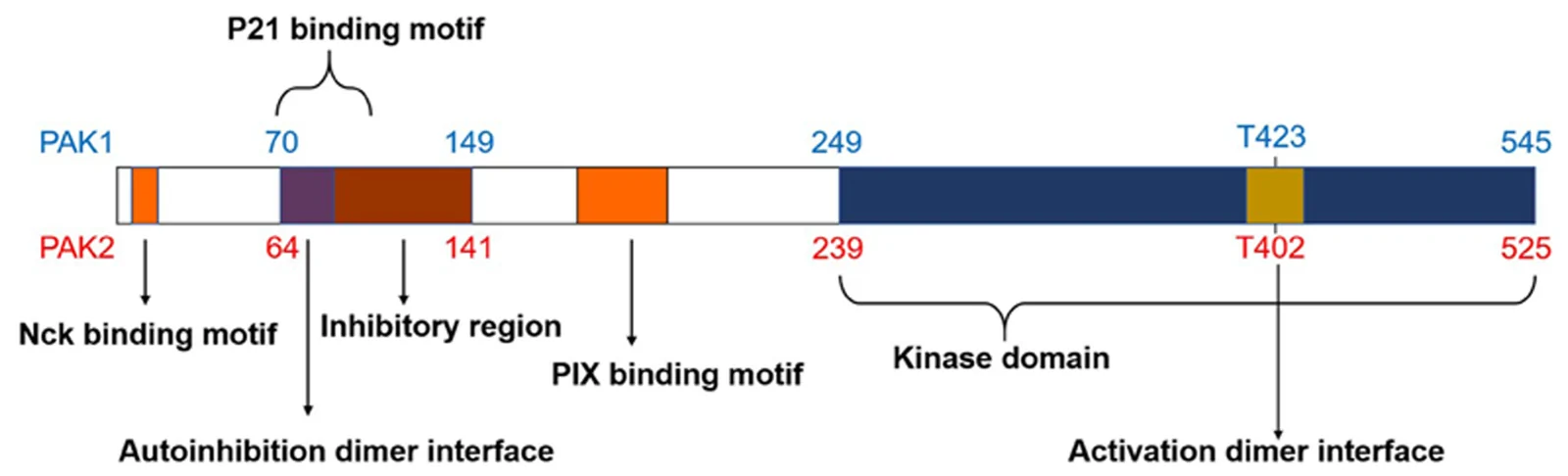
Structural domains of Pak1 and Pak2. Nck and PIX are the two SH3-binding domains (orange); the autoinhibition dimer interface (purple) overlaps with the p21-binding domain and inhibitory region (AID) (brown); the kinase domain is at the C-terminus (blue); activation dimer interface is within the kinase domain (yellow).

Pak1 and Pak2 are highly conserved proteins with multiple SH3-domain binding sites which belong to the Pak protein group I [8] (Figure 1). Pak1 (α-Pak) is a 68 kDa protein mainly expressed in the brain tissues, muscles, spleen and the heart [9]. Molecular cloning and sequencing have revealed high homology of Pak1 cross the human placenta, rat brain, and STE20 yeast [10]. Pak1 and Pak2 Share a 91% sequence homology identity within their kinase domains and they are ubiquitously expressed in various cell types [11]. The non-catalytic region of tyrosine kinase adaptor protein (Nck) and Pak-interacting exchange factor (PIX) are crucial binding sites as they contain proline-rich regions that directly bind to specific proteins, thus promoting the translocation and activation of downstream proteins [12, 13]. The N-terminus autoinhibitory domain interacts with p21 protein and plays a crucial role in Pak activation [14]. The C-terminus kinase domain is highly conserved in group I and II Paks. Paks also contain multiple conserved phosphorylation sites. Although the majority of these phosphorylation sites are serine points, an important threonine site (T423 in Pak1 and T402 in Pak2) is present in the kinase domain and it is a critical indicator of Pak activation [14,15].

Due to their conserved structure, Pak group I proteins have similar activation mechanisms. Structural studies have shown that group I Paks are activated via a trans-auto-inhibition mechanism, and inactive Pak exists as a dimer [14]. In the inactive state, Pak inhibitory region interacts with the kinase domain. Such interaction inhibits the auto-phosphorylation of the kinase region. Activated GTP-bound Rac1 binds to the p21-binding region to induce a conformational change of Pak. This results in the autophosphorylation of several corresponding sites. Ser144 phosphorylation within the inhibitory region (Figure 1) promotes dimer dissociation to expose the kinase domain to allow autophosphorylation. Phosphorylation of Thr423 activates Pak 1, further promoting the phosphorylation of the downstream targets [14].

### 1.2 Pak1 Activation and Signalling Pathways

The first Pak protein to be discovered and characterised was Pak1. It was found to modulate cell cytoskeleton reorganization and motility as well as endothelial cell migration [16]. Pak1 has also been shown to play a role in numerous pathological conditions, such as cardiovascular disease, neurodevelopmental disorder, and cancer [6,17,18]. Evidence from prior studies has demonstrated that Pak1 participates in numerous signalling cascades and cellular events.

In addition to the Rho GTPase-mediated activation, Pak1 activity can be regulated through other mechanisms. For instance, it has been reported that lipids such as sphingolipids can contribute to Pak1 activation in the absence of GTPase [19]. Lipids act on an overlapping site in the GTPase-binding domain of Pak1 [19]. The inactive sphingosine derivative has been found to inhibit Pak1 activation in response to sphingosine or Cdc42 [19].

Pak1 transmits signals from the cell membrane to the cell cytoplasm by interacting with Nck adaptor protein, and binding to the SH3 motif near the N-terminus (Figure 1) [20]. Activation of Pak1 by the epidermal growth factor receptors (EGFR) and platelet-derived growth factor receptors (PDGFR) [21] triggers the direct interaction of Nck with the Pak1 SH3 motif on Pak proteins [20].

The mitogen-activated protein kinase (MAPK) cascade is one of the well-characterised downstream pathways of Pak1. MAPK has been implicated in the regulation of several processes including cell differentiation, apoptosis, motility and metabolism [22]. The main three MAPKs involved in Pak signalling are the extracellular signal-regulated kinase (ERK), p38, and c-Jun N-terminal kinases (JNK). Ras is an upstream protein of the Raf/MEK/ERK pathway belonging to the MAPK signalling cascade. In an earlier study, expression of catalytically inactive mutant Pak1 in rat fibroblast cells inhibited Ras transformation and ERK activation [23]. As previously mentioned, Pak1 is the human homology of the yeast Ste20p protein [10]. Ste20p activates MEKK (MAPKKK), which further activates MEK (MAPKK) independent of the Ras-Raf-MEK pathway [24]. *In vitro* experiments have shown that Pak1 can activate the p38 and JNK pathways, the members of the MAPK family [25]. Interleukin-1 (IL-1) activates Pak1 via GTPases Cdc42 and Rac1, which further induces p38 activation. Pak1 directly phosphorylates Raf and Mek1 in human cells. Pak1 phosphorylates c-Raf at Ser338 and Mek1 at Ser298 [26]. Pak1 inhibition attenuates Mek1 phosphorylation through EGFR and PDGFR pathways, showing the importance of Pak1 on growth factor receptor activity.

Pak1 affects cell mitosis by regulating the centromere duplication [27]. The centrosomal adaptor GIT1 directly interacts with Pak1 via the PIX region (Figure 1). Activated Pak1 is recruited to GIT1 through the SH3 PIX domain, phosphorylating GIT1 at the Ser517 site [28]. In addition, Pak1 regulates mitosis involves the activation of Pak1 by centrosomes in a Rho GTPases-independent manner. This process allows the dissociation of Pak1-PIX-GIT1 and promotes the phosphorylation of centrosomal kinase Aurora-A at Thr288 and Ser342; these two sites are crucial for mitotic kinase activation [28]. These specific mechanisms demonstrate the role of Pak protein family in mitosis.

Pak1 has also been found to participate in inflammatory processes. Activated Pak1 induces the activation of NFκB in macrophages [29]. As mentioned earlier, Pak1 is not only activated by the Rho GTPases but also by other molecules, such as lipids. In macrophages, lipopolysaccharides activate Pak1 to stimulate p65 subunit of NFκB [29]. Pak1-mediated nuclear translocation of p65 further activates NFκB but not IKKβ, an inhibitor of NFκB [29]. Pak1 is also involved in autophagy. Hypoxia-induced Pak1 acetylation may phosphorylate ATG5 at the T101 residue and promote autophagosome formation [30].

The anti-apoptotic role of Pak1 have also been well studied. Pak1 induces Raf1 phosphorylation at Ser338 and translocation to mitochondria, leading the formation of the Raf1-Bcl-2 complex to prevent apoptosis in HEK293T cells [31]. In addition, Pak1 phosphorylates the pro-apoptotic protein Bad at Ser112 and inhibits its interaction with Bcl-2, thereby protecting lymphoid progenitor cells from apoptosis [32]. Another study also reported that Pak1 knockdown enhances apoptosis in hepatocellular carcinoma cells via the p53/p21 signalling pathway [33]. The pro-survival role of Pak1 has also been observed in β-cell. β-cell-specific Pak1 knockout mice exhibited impaired redox imbalance due to mitochondrial dysfunction, which led to the generation of ER stress and β-cell apoptosis [34]. Pak1 overexpression in the type 2 diabetes human islet β-cells ameliorates ER stress and improves β-cell function [34]. Considering the important role of Pak1 in cell survival pathways, it holds great promise in the treatment of cardiovascular disease treatment. However, additional mechanisms may be involved, and the effect of Pak1 inhibition on apoptosis of cardiomyocytes needs to be further investigated.

### 1.3 Pak2 Signalling Pathways

Pak2 is another isoform belonging to group I Pak family of proteins, sharing high sequence similarities with Pak1, suggesting some common activation mechanisms between Pak1 and Pak2 [35]. For example, Pak2 can also interact with GIT1 through the Pak/PIX/GIT1 complex to regulate epithelial cell migration [36]. Numerous stressors can activate Pak2, such as serum starvation, DNA damage and hyperosmolarity [37–39]. Previous studies reported that Pak2 may have dual functions on cell survival following exposure to different types of stress [40]. Pak2 can be activated by cdc42 in full-length form to mediate survival pathways [41,42]. In addition, oxidative stress can induce caspase-3 to cleave full-length Pak2 into two fragments, the p27 N-terminal regulatory domain and the p34 C-terminal catalytic domain, which triggers apoptosis [43]. A recent study demonstrated that Pak2 showed resistance to caspase-3-induced cleavage following activation by cdc42, and it inhibited oxidative stress-induced apoptosis in mammalian cells [40]. Given that researchers used forced Pak2-p34 or a caspase resistant form Pak2-D212N expression in mouse fibroblasts and HEK293T, whether Pak2-p34 has physiological functions that remain to be clarified. We speculate that cleaved Pak2 may exist as a transient state before proteasomal degradation.

Pak2 is involved in the regulation of contractility of smooth muscle cells and non-muscle cells [44–47]. Activated Pak2 phosphorylates non-muscle myosin II on the myosin regulatory light chain (RLC) [48]. A phosphopeptide map has demonstrated that Ser19 is the only phosphorylation site on RLC regulated by Pak2. Activated Pak2 and RLC are important for cell retraction and actin rearrangement, implying that Pak2 may participate in the regulation of cytoskeletal organization [48]. Pak2 also modulates the phosphorylation of myosin light chain kinase (MLCK). MLCK is a crucial kinase that phosphorylates RLC to enhance the contractile function of myosin II. Pak2 catalyses MLCK phosphorylation at Ser439 and Ser991, which prevents myosin II RLC phosphorylation [46].

Pak2 negatively regulates glucose uptake in neuronal cells [49]. Studies have demonstrated the association between Pak2 activation and the expression of protein phosphatase 2A (PP2A) in neuronal cells. Inhibition of PP2A enhances Pak2 activity and vice versa. In addition, over-expression of Pak2 modulates glucose uptake and GLUT4 translocation, whereas downregulation of Pak2 triggers opposite effect [49].

In endothelial cells, Pak2 was found to regulate Erk5 to maintain endothelial development and function [50]. Erk5 regulates fatty acid oxidation by upregulating the expression of peroxisome proliferator-activated receptor γ co-activator-1α (Pgc-1α) [51], suggesting that Pak2 may regulate mitochondrial function through this pathway. Pak2 enhances Smad2/3 activation and transcriptional responsiveness in MDCK epithelial cells [52]. Moreover, the activation of the TGF-β/Smad/METTL3 pathway upregulates Sec62 expression to promote autophagy [53,54]. Although this mechanism has not been demonstrated in cardiomyocytes, these findings suggest a potential role of Pak2 in autophagy. Pak2 also participates in inflammation. In the diabetic mouse model, Pak2 deficiency in the heart aggravates inflammation by increasing the expression of CCAAT/enhancer-binding protein homologous protein (CHOP) and high-mobility group box-1 (HMGB1), which promotes M1 macrophage polarization [55].

**Figure 2:**
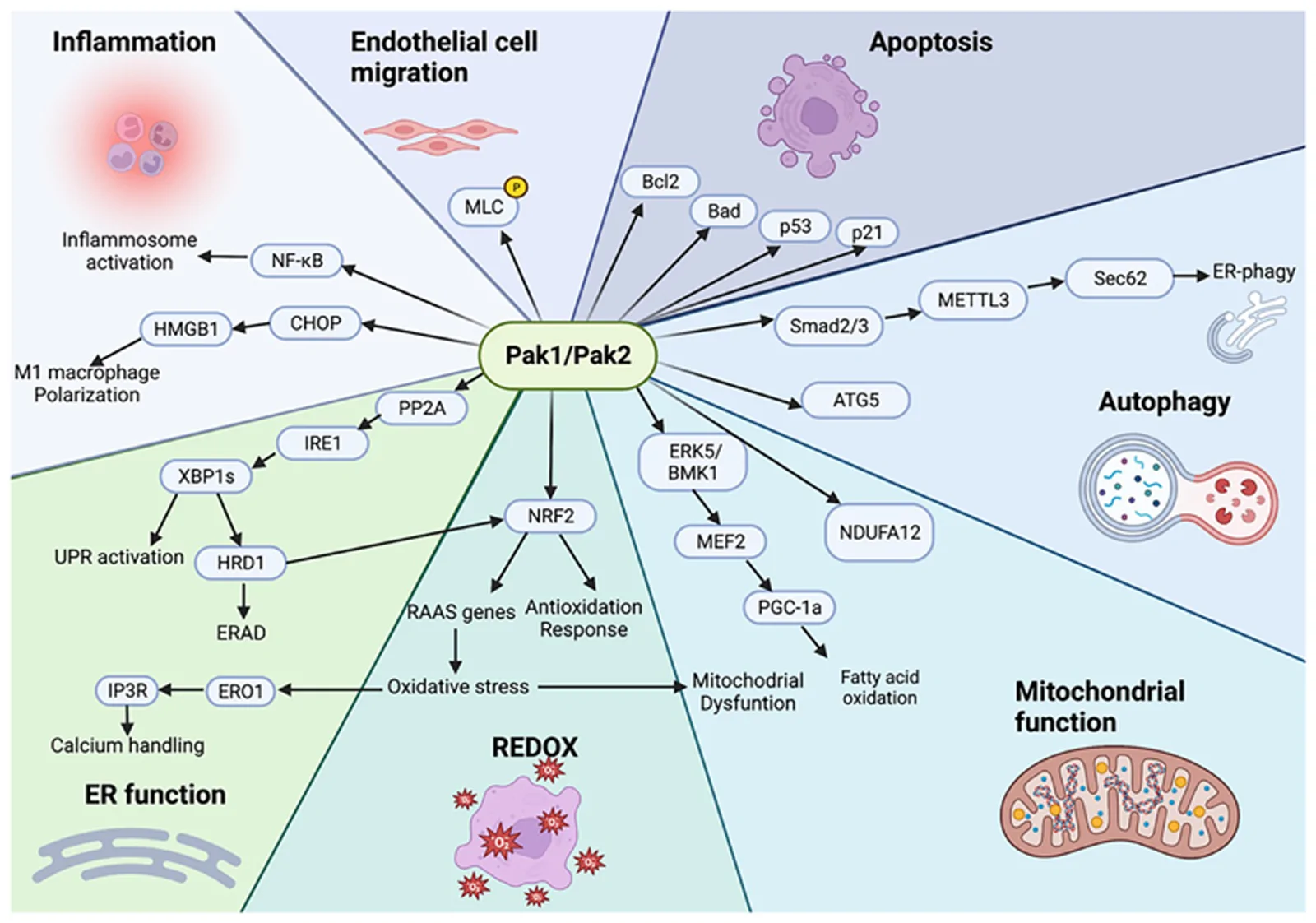
The signalling nexus of Pak1 and Pak2. Mechanisms underlying the multiple physiological effects of Paks. Pak1/2 phosphorylate myosin light chain (MLC) to regulate endothelial cell migration and contractility. Pak1 phosphorylates Bad to inhibit its interaction with Bcl2 to prevent apoptosis. Pak1 exerts anti-apoptotic effects via the p53/p21signalling pathway. Pak1 acetylation can phosphorylate ATG5 to promote autophagosome formation. Pak2 enhances Smad2/3 activation and upregulates Sec62 expression to promote autophagy. Pak1 regulates NDUFA12 expression level to modulate mitochondrial function. Pak2 regulates fatty acid oxidation and mitochondrial homeostasis by acting through the ERK5/PGC-1α pathway. Pak2 regulates Nrf2 expression and the subsequent ROS production. Pak2 maintains ER function and proteostasis via the IRE1/XBP1 signalling pathway. Pak1 activates NFκB to influence immune responses. Pak2 regulates M1 macrophage polarization to play anti-inflammatory roles. ER: Endoplasmic Reticulum; REDOX: Reduction-Oxidation.

Considering that Pak1/2 proteins are involved in various cellular events, and many of which play crucial roles in cardiovascular disease progression (Figure 2), a better understanding of the mechanisms regulated by Pak1/2 in the heart is needed to promote translational research of molecules targeting these protein kinases.

### 2.1 Role of Pak1 in Cardiovascular System

#### 2.1.1 Pak1 in the Development of Atherosclerosis

Atherosclerosis is characterized by thickening or stiffening of large arteries, and it is caused by the formation of atherosclerotic plaques in the inner lining of blood vessels. Multiple cellular and molecular events drive atherosclerotic lesion formation. Inflammation, high permeability of the endothelium lipids, vascular remodelling, macrophage recruitment, and cell turnover contribute to the development of atherosclerosis. Pak1 expression and activity are increased in atherosclerosis-prone apolipoprotein E-deficient (ApoE^-/-^) mice. Similarly, increased Pak1 phosphorylation was also observed in human atherosclerotic arteries, suggesting that Pak1 plays a significant role in the development of atherosclerosis [56].

As described in the previous section, Pak1 participates in the regulation of inflammation. Macrophages play key roles in inflammation during the development of atherosclerosis. Pak1 expression was reported to upregulate in M1 macrophages but downregulate in M2 macrophages in isolated bone marrow-derived macrophages and peritoneal macrophages of ApoE^-/-^ mice [57]. It was also reported that Pak1 knockdown led to increase the anti-inflammatory M2 macrophages activation markers expression (IL-10, arginase-1) and decrease the pro-inflammatory M1 macrophages activation markers expression (IL-6, IL-1β) [57]. The changes in the expression of the aforementioned markers upon Pak1 silencing were mediated by endogenous interaction between Pak1 and PPARγ [57]. Moreover, Pak1 acts on different inflammatory pathways in the endothelium, including NF-κB and JNK [58,59]. *In vivo* experiments demonstrated that depletion of Pak1 in C57BL/6J mice decreased the expression NF-κB (p65 subunit), implying that Pak1 promotes inflammation at atherosclerosis sites [60]. Furthermore, prior studies have demonstrated that nicotinamide adenine dinucleotide phosphate-oxidase (NADPH oxidase) participates in the initiation and progression of atherosclerosis, and Pak1 is implicated in the regulation of NADPH oxidase (NOX2) activity in diseases associated with inflammation activation [61, 62]. However, the regulation of NADPH oxidase by Pak1 in the context of inflammation during atherosclerosis is yet to be experimentally verified.

#### 2.1.2 Pak1 in Hypertrophy

Cardiac hypertrophy is an adaptive response to hemodynamic stress and can cause heart failure. Cardiac hypertrophy is characterized by the proliferation of cardiac fibroblasts, hypertrophic growth of cardiomyocytes, as well as increased deposition of extracellular matrix (ECM) constituents, all of which alter the cardiac function. Pak1 can be activated by various hypertrophic agonists including angiotensin II (Ang II), phenylephrine, and isoprenaline [6]. Pak1 phosphorylation was increased in cardiomyocytes of mice subjected to transverse aortic constriction (TAC) for 2 weeks [6]. Moreover, cardiomyocyte-specific Pak1 deletion (Pak1-CKO) mice developed significant cardiac hypertrophy accompanied with increased fibrosis and cardiomyocyte size compared to control mice after two weeks of pressure overload stress [6]. Apart from pressure overload stress-induced cardiac hypertrophy, Pak1 also protects the heart from neuroendocrine agonist-induced hypertrophic stimulation. Pak1-deficient mice displayed increased susceptibility to Ang II or isoprenaline mediated cardiac hypertrophy [6,63,64]. These results suggest an anti-hypertrophic role of Pak1 in the heart. In the Pak1-CKO mouse model, the phosphorylation of MKK4, MKK7 and JNK was not increased as seen in control mice after TAC surgery [6]. This indicates that Pak1 protects the heart from hypertrophic stress by stimulating MKK4/MKK7-JNK pathway. On the other hand, ERK1/2 activation was increased in Pak1-CKO mice with isoprenaline-induced hypertrophy [64]. This suggests that Pak1 suppresses ERK1/2 activation in isoprenaline-triggered hypertrophy to confer cardioprotection.

#### 2.1.3 Pak1 in Ischemic/Reperfusion (I/R) Injury

Restoring blood flow to the ischemic myocardium is one of the most common treatment strategies for ischemic heart disease. Re-establishment of blood supply reduces infarct damage and the risk of death. However, sudden or inadequate coronary perfusion may cause reperfusion injury, leading to a series of consequences, including cardiomyocyte death and cardiac arrest [65,66]. FTY720 is a synthetic sphingosine analogue that activates Pak1 to protect against cardiac ischemia/reperfusion (I/R) injury in animals [67, 68]. PI3K activated Pak1 to alleviate the detrimental effects of I/R on heart function by inhibiting cardiac myocyte apoptosis through the PI3K/Akt signalling pathway [69]. I/R injury increased cardiac troponin T (TnT) isoforms-TnT3 and TnT4 phosphorylation levels in wild-type mice, but these events were not observed in Pak1-KO hearts after I/R injury [70]. Moreover, Pak1 knockout exacerbated the ventricular performance after I/R injury [70]. Together, these suggest that Pak1 regulates the expression of myofilament proteins and alleviates I/R injury.

#### 2.1.4 Pak1 in Arrhythmia

Significant progress has been made in understanding the role of Pak1 and Ca^2+^ homeostasis in the pathogenesis of cardiac arrhythmias. Deficiency of Ca^2+^ promotes the pathogenesis of arrhythmia [71]. *In vivo* and *in vitro* studies have shown that Pak1 maintains cardiac excitation and contraction dynamics by regulating ion currents. Activation of Pak1 increased Ca^2+^ sensitivity and improved the function of downstream proteins, such as L-type Ca^2+^ channels, Ca^2+^-ATPase 2a (SERCA2a), and sarcolemma reticulum (SR) [72]. In Ang II-treated ventricular myocytes, the amplitudes of calcium transients were significantly reduced, and the peak-plateau duration was significantly prolonged [63]. In contrast, Ang II significantly increased Ca^2+^ sparks and waves in myocytes, however, this effect was reversed following treatment with Pak1-activating peptide, implying that Pak1 alleviates the detrimental effect of Ang II on Ca^2+^ handling [63]. *In vitro* studies indicated that Pak1 deficiency disrupted Ca^2+^ homeostasis, especially during isoprenaline-induced chronic β-adrenoceptor mediated stress in cardiomyocytes. Pak1 knockout decreased Ca^2+^ transient amplitude and delayed action potential repolarization in isolated ventricular myocytes [73]. In Pak1-CKO mice, SERCA2a expression was altered, which in turn changed Ca^2+^ homoeostasis [74]. Pak1 regulates SERCA2a expression probably through the serum response factor (SRF) [75]. In addition, Pak1 overexpression reduced cTnI phosphorylation at Ser23/24, thereby improving myocyte contractility kinetics [76].

Loss of Pak1 promotes the inducibility of arrhythmia. Jaime DeSantiago et al. reported that loss of Pak1 in ventricular myocytes enhanced NADPH oxidase 2-mediated ROS production and exaggerated the increase in cytosolic Ca^2+^ concentration [Ca^2+^]_i_ through elevating sodium-calcium exchanger (NCX) activity [77]. This mechanism was further explored in atrial myocytes which showed that inhibition of Pak1 activity promoted atrial arrhythmia [78]. Moreover, Pak1 stimulation by FYT720 prevented the occurrence of arrhythmic events in atrial myocytes from a canine model of atrial fibrillation [78]. In addition, a recent study showed that Pak1 modulated small-conductance Ca^2+^-activated K^+^ (SK) 2 channel [79]. SK2 channel is activated by submicromolar [Ca^2+^]_i_, which then affects cardiomyocyte membrane potential and cardiac arrhythmogenic tendency under cardiac hypertrophy and/or heart failure condition [79–82]. In Ang II-induced hypertrophy mouse model, knockout or knockdown of Pak1 accentuated the increase of SK2 channel current density and protein expression, while stimulation of Pak1 by FYT720 mitigated these effects [79]. Together, these studies demonstrated the anti-arrhythmic benefits of Pak1.

### 2.2 The role of Pak2 in Cardiovascular System

To date, evidence emerging from recent studies has indicated that Pak2 prevents the development of several cardiovascular diseases [7,55,83]. Pak2 can regulate angiogenesis and endothelial cell survival. The prosurvival role of Pak2 was first observed in zebrafish^84^. Loss-of-function of the *pak2* gene was associated with defects in endothelial cells and contributed to haemorrhage [84]. These findings suggest that Pak2 improves the functional integrity of vascular tissues. In addition, global Pak2 deletion resulted in foetal death by inducing multiple developmental abnormalities, mainly associated with vascular defects in mice [50]. In adult mice, endothelial Pak2 deletion promoted vascular permeability, suggesting that Pak2 is an important regulator of development and maintenance of endothelial cell function [50].

As the largest cellular organelle, the endoplasmic reticulum (ER) plays an essential role in protein synthesis and folding. It regulates several transcriptional and translational programmes, calcium homeostasis, lipid, and steroid biosynthesis, as well as modulating protein translocation [85]. Unfolded protein response (UPR) of ER modulates cellular homeostasis through three transmembrane stress sensors: inositol-requiring enzyme 1 (IRE1), protein kinase-like ER kinase (PERK), and activating transcription factor 6 (ATF6) [86]. The ER stress plays a central role in the development of cardiovascular disease. It can activate adaptive UPR to accelerate protein folding and ensure protein quality control. However, excessive ER stress damages protein folding capacity and switches ER stress response to the detrimental phase [87]. Pak2 maintains UPR and improves the ER function in the heart. In the TAC induced hypertrophic mouse model, Pak2 battled ER stress by suppressing the PP2A activity to preserve IRE1 phosphorylation and stimulated the IRE1/XBP1 signalling pathway. The Pak2 activity under stress condition was also changed in a time-dependent manner. During the acute stage of ER stress, Pak2 was activated triggering adaptive UPR and restoration of ER homeostasis, leading to normalization of heart function. In the late stage of ER stress, Pak2 was inactivated resulting in cardiac dysfunction^7^. Unlike global Pak2 knockout that causes foetal death, cardiac-specific loss of Pak2 has no effect on cardiomyocyte function under physiological state [7,50]. However, during pressure overload or metabolic stress condition, loss of Pak2 in the heart impaired the activation of the ER stress response causing cardiomyocytes dysfunction and heart failure. Moreover, Pak2-regulated ER stress response was also observed under hypoxia-reoxygenation [88], as well as ischemia/reperfusion injury [73]. Collectively, these findings confirm that Pak2 confers cardioprotective effects in response to several types of stresses.

Notably, studies have shown that Pak2 plays other roles besides regulating ER function in the heart. Pak2 regulates the expression of Nrf2 and serves as a signalling nexus connecting the renin-angiotensin-aldosterone system (RAAS) activation, oxidative stress and UPR. In the Pak2 knockout heart, impaired UPR response led to excessive Nrf2 expression, promoted ROS production and RAAS genes overexpression during pathological condition, thus resulting in increased cardiomyocyte death [83]. Nrf2 is normally served as a key transcription factor regulating the expression of antioxidant and detoxification genes to protect the heart from oxidative stress injury [89]. However, previous studies have shown that Nrf2 has a detrimental effect on the heart [90–92]. In the transgenic mice with sustained Nrf2 activation in the heart, increased protein aggregation, pathological hypertrophy and heart failure were observed [90]. Nrf2 deficiency protects the mouse heart against ischemia-reperfusion (I/R) injury by upregulating nitric oxide (NO) production [91]. Moreover, in mice having insufficient autophagy during cardiac maladaptive remodelling, Nrf2 knockout exerted cardioprotective effects by preventing increased expression of autophagy related gene 5 (Atg5) in response to pressure overload stress [92]. Although precise mechanisms underlying Nrf2-mediated cardiac damages are unclear, it is postulated that Pak2 phosphorylation is decreased during long term stress, which impairs UPR to cause defective proteostasis in cardiomyocytes. This pathological event may contribute to aberrant activation of Nrf2.

A recent study using mice fed with high-fat diet induced-cardiomyopathy demonstrated that Pak2 also participated in inflammatory responses to metabolic stress [55]. Pak2 deletion in the diabetic heart increased CHOP, which directly upregulated HMGB1 protein expression [55]. Activated HMGB1 in the diabetic heart promoted the polarization of bone marrow-derived macrophages to the M1 subtype, resulting in detrimental inflammatory reactions in the myocardium [55]. In addition, the expression of ATF4 and ATF6 was upregulated whereas that of XBP1s was downregulated in the myocardium of diabetic mice and patients, suggesting that Pak2 deletion exacerbates ATF4 and ATF6-mediated increase in CHOP expression [55]. Of note, CHOP, a downstream target of ATF4 and ATF6, regulates the pro-apoptotic pathway during ER stress signalling. These findings demonstrate that the maladaptive ER stress in the Pak2 deficiency heart triggers the upregulation of CHOP expression and aggravates inflammatory responses, suggesting that Pak2 prevents inflammation in the myocardium under metabolic stress.

In aggravate, Pak2 deficiency-induced ER dysfunction may be a key cellular event promoting inflammation and ROS generation. Therefore, therapeutic strategies modulating Pak2 activation to maintain cardiac ER function are worth exploiting as new means to treat cardiovascular disease.

## 3 The Therapeutic Values of Pak1 and Pak2 in Cardiovascular Disease

The participation of Paks in diverse biological processes in the heart suggests their board values in translational exploitations. Given the role of Pak1/2 in the cardiovascular system, strategies targeting Pak1 and Pak2 could be promising avenues for treating cardiovascular disease by maintaining cellular homeostasis, metabolic function, and enhancing cardiomyocyte adaptation and resilience to stress.

The Vaughan-Williams classification system is the most commonly used system to classify anti-arrhythmic drugs. A recent publication of the modernized version of the Vaughan-William classification system proposed that novel molecular targets related to Ca^2+^ homeostasis could be classified into Class IV [93]. Based on the significant role of Pak1 in the regulation of cardiac Ca^2+^ homeostasis, it may become a novel anti-arrhythmic therapeutic target.

Sphingosine-1-phosphate (S1P), a bioactive sphingolipid metabolite that activates Pak1 has been reported to protect cardiomyocytes from I/R injury [94–96]. Fingolimod (FTY720) is an FDA-approved sphingolipid drug with a similar structure to S1P, it exerted cardioprotective effects against I/R injury and hypertrophy [6,63]. Similarly, FTY720 also prevented the arrhythmias occurrence by regulating the Pak1/Akt signalling pathway in rat hearts subjected to I/R injury [67]. FTY720 stimulated NO production via the PI3K/Akt/eNOS signalling pathway to prevent hypoxic/ischemic cell injury [97]. Mice having TAC-induced cardiac hypertrophy were administered with FTY720 and exhibited a reduction in pathological cardiac hypertrophy [98]. These findings suggest that FTY720 can prevent cardiac hypertrophy and I/R injury. Thus, Pak1 activation holds a great promise in the treatment of cardiovascular disease.

Cardiomyocytes have a low proliferative capacity. Therefore, loss of cardiomyocyte function during ER stress ultimately causes heart failure and death. A study in zebrafish revealed that Rac1-mediated activation of Pak2 could regulate cardiomyocyte proliferation [99]. Heart regeneration involves numerous processes including epicardium regeneration, angiogenesis, inflammatory responses, and cardiomyocyte proliferation [100, 101]. This indicates that modulating Pak2 could be a feasible approach for regulating cardiomyocyte proliferation at some extent used for heart failure treatment.

In the TAC mouse model, Pak2 overexpression was able to maintain ER homeostasis and protect against heart failure by inhibiting apoptosis and improving cardiac function^7^. Similarly, Pak2 was also found to facilitate ER function in the human cardiomyocytes [7]. Involvement of Pak2 in the ER regulatory mechanism suggests that Pak2 can be targeted to develop ER-orientated interventions against cardiovascular disease. Vildagliptin, an anti-diabetic drug, was reported to restore Pak2 activity and alleviate ER stress-induced inflammation in the diabetic mouse model [55]. However, it was demonstrated that prolonged metabolic stress decreased Pak2 expression and activity, which suppressed the anti-inflammation effect of vildagliptin [55]. This may explain the limited benefits of vildagliptin in improving cardiac function in clinical trails [102]. Therefore, anti-inflammatory effects of Pak2 can be leveraged to develop potential therapeutic strategies for treating myocardial inflammation involved diseases.

Evidence has demonstrated that Pak1/2 are upregulated in human tumour tissues, such as breast cancer, gastric cancer, ovarian cancer, and head and neck cancer [103–106]. Prior investigations have shown that Pak1/2 promote the proliferation, survival and invasion of tumour cells [107–109]. Although they exert cardioprotective effects, the association of Pak1/2 with cancer could limit their cardiovascular applications. To address safety concerns on developing Pak1/2 activating drugs, new technologies such as nanoparticles or adeno-associated virus (AAV) for precise delivery in the heart and vasculature sites could be used.

## 4 Conclusion

Since the first identification of Pak1 in 1994, several types of Paks, their structures, functions, and activation mechanisms have been gradually discovered. The majority of previous studies were focused on investigating the therapeutic benefits of Paks inhibition in cancers [61]. Over recent years, experimental evidence has shown that Pak1/2 exert cardioprotective effects. The expression of Pak isoforms varies among tissues and organs, and their subcellular localisations and functions also differ in a tissue/organ-specific manner. Therefore, future studies are needed to develop strategies that can target specific Pak1/2 motifs and their intracellular microdomains to confer cardioprotective effects.
